# Genomic Variation and GWAS Analysis for Salt Tolerance Discovered in Egyptian Rice Germplasm

**DOI:** 10.3390/plants15010128

**Published:** 2026-01-01

**Authors:** Yueying Wang, Faming Yu, Sirinthorn Kongpraphrut, Congcong Liu, Muhammad Asad Ullah Asad, Salma Kelany, Mengrui Sun, Yuxuan Wang, Yang Lv, Galal Anis, Mohamed Hazman, Qian Qian, Yuexing Wang, Longbiao Guo

**Affiliations:** 1 State Key Laboratory of Rice Biology and Breeding, China National Rice Research Institute, Hangzhou 310006, China; wyywangyueying@163.com (Y.W.); sirinthorn.k@rice.mail.go.th (S.K.); lcc43932@163.com (C.L.); salmakelany93@gmail.com (S.K.); 15864914259@163.com (M.S.); yuxuan_wang21@163.com (Y.W.); qianqian188@hotmail.com (Q.Q.); 2 Institute of Crop and Nuclear Technology Utilization, Zhejiang Academy of Agricultural Sciences, Hangzhou 310021, China; yfm4679@163.com (F.Y.); asad.gu@hotmail.com (M.A.U.A.); lvyang0725@163.com (Y.L.); 3 Krabi Rice Research Center, Rice Department, Krabi 81130, Thailand; 4 Rice Research and Training Centre, Field Crops Research Institute, Agriculture Research Center, Kafrelsheikh 33717, Egypt; galalanis@arc.sci.eg; 5 Agricultural Genetic Engineering Research Institute, Agricultural Research Center, Giza 12619, Egypt; yousof.hazman@outlook.com

**Keywords:** salt tolerance, Egyptian rice, genomic divergence, GWAS, haplotype

## Abstract

Egyptian rice landraces represent a unique genetic reservoir shaped by arid environments, yet their genomic and transcriptional response to salt stress remains largely unexplored. Here, we integrated genomic, transcriptomic, and population genetic analyses to systematically unravel the mechanisms of salt tolerance in this vital germplasm. Resequencing 56 Egyptian accessions uncovered a treasure trove of genetic variation, including 18,204 novel SNPs. An expanded GWAS on 258 accessions discovered 17 novel loci for salt tolerance. Parallel RNA-Seq analysis of a salt-tolerant-susceptible pair (Giza 176 vs. 9311) under stress delineated a defense network centered on phenylpropanoid and lipid metabolic pathways in the tolerant genotype. The power of our integrated approach was exemplified by the convergent identification of *ONAC063*, where GWAS loci, transcriptional responsiveness, and haplotype-phenotype association collectively validated its role. Furthermore, selection sweep analysis highlighted 62 candidate genes under divergent selection. Our study not only positions Egyptian rice as a key resource for allele mining but also establishes a robust multi-omics pipeline for bridging genetic diversity with complex traits, accelerating the discovery of functional genes for breeding climate-resilient crops.

## 1. Introduction

Rice (*Oryza sativa* L.) is a staple food for nearly half of the world’s population, making the stability of its production crucial for global food security [1]. Soil salinization represents a major constraint on sustainable rice cultivation, whose exacerbation is closely linked to irrational human agricultural practices and natural elements [2]. Rice is a crop that is relatively sensitive to salt stress. There are significant differences in salt tolerance among different rice varieties and across various growth stages, with the early seedling and reproductive stages being the most sensitive [3,4]. Rice grown in saline soil often exhibits physiological metabolic disorders, which in turn lead to reduced biomass and severe yield losses [5]. Excessive soluble salts in soil impair photosynthesis, induce ionic toxicity and oxidative stress in rice, resulting in a substantial reduction in panicle number, grain number, and thousand-grain weight [6]. The rice germplasm resources exhibit abundant genetic variation in salt tolerance, providing a basis for breeding improvements. Enhancing the salt tolerance of rice to boost yields in saline-alkali fields is a key strategy to tackle the global soil salinization challenge and ensure food security [7].

The plant salt stress response involves a complex set of physiological and biochemical processes [8]. Stress sensor is the first response of plants to abiotic stress, and it is an effective adaptation strategy of plants to the external environment. Under salt stress conditions, osmotic and ionic stress are sensed by membrane-bound cytosolic sensors that ultimately trigger early salt stress signaling pathways [9]. Stress signals are regulated by rapid and long-distance signaling processes, including phospholipid-based signaling, mitogen-activated protein kinases (MAPK), reactive oxygen species (ROS) and abscisic acid (ABA), calcium signaling, and long-range electrical signaling [10,11]. Under osmotic stress, activated sensory factors promote the production of tocopherols and related metabolites. This response serves to lower water potential, which in turn minimizes the loss of intracellular water. In addition, a variety of ion transporters and channel proteins, including High-affinity K^+^ Transporters (HKT), Na^+^/H^+^ Exchangers (NHX), and High-affinity K^+^ transporters (HAK), are involved in rebuilding ion balance in plants and regulating tolerance to high salts [12,13]. However, when the salt concentration transcends the plant’s tolerance threshold, the plant fails to sustain K^+^/Na^+^ homeostasis within cells, giving rise to ion toxicity.

Rice salt tolerance, a quintessential quantitative trait with an intricate polygenic inheritance, has been the focus of extensive genetic mapping efforts [14]. To date, more than a thousand genes/QTLs associated with various salt tolerance-related traits (e.g., survival days, ion homeostasis, stomatal aperture) across different developmental stages have been identified [15]. The shift towards genome-wide association studies (GWAS), leveraging high-density SNP chips and linkage disequilibrium, has marked a significant advancement, offering superior resolution and power in allele detection for complex traits [16,17]. This methodology has been successfully applied to dissect rice salt tolerance, yielding numerous associated loci from studies involving hundreds of diverse accessions [18,19]. The resulting QTLs have catalyzed the functional validation of key genes, including *DST* [20], *OsHKT1;5* [21], and *RST1* [22]. Concurrently, breakthroughs in sequencing technology have facilitated gene cloning via transcriptomic analyses in structured populations, exemplified by the discovery of *STG5* [23]. Ultimately, deciphering the distinct genetic mechanisms underpinning salt tolerance across varied rice germplasm is crucial for developing resilient cultivars.

Egyptian rice germplasm, evolved under a tropical desert climate, is a potential source of unique alleles for salt tolerance, yet the genetic basis of this trait is poorly characterized. To systematically decipher the adaptive mechanisms, we implemented an integrated genomic and transcriptomic approach. This study combines GWAS on a diverse panel of 56 Egyptian and 202 Asian rice accessions with comparative RNA-Seq analysis of tolerant and sensitive cultivars under salt stress. This strategy enables us to not only map genomic loci associated with tolerance but also to validate and prioritize candidates within functional transcriptional networks, thereby identifying novel genes for the molecular breeding of resilient rice.

## 2. Results

### 2.1. Population Structure Analysis

**Figure 1:**
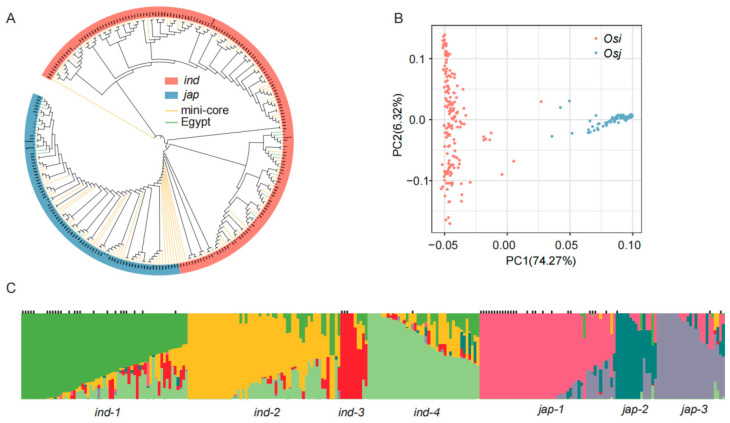
Population structure analysis. (**A**) Principal component analysis; (**B**) phylogenetic tree; (**C**) population structure distribution.

To elucidate the population structure of 56 Egyptian rice accessions, we integrated their genomic data with 202 publicly available Asian cultivated rice genomes. A Neighbor-Joining phylogenetic tree constructed from this combined dataset of 258 accessions revealed two primary clades corresponding to the *indica* (*Osi*) and *japonica* (*Osj*) subspecies. The Egyptian accessions were equally distributed between these two lineages, with 28 accessions clustering within the *indica* branch and the remaining 28 within the *japonica* branch, indicating their distinct genetic affiliations with these major subgroups (Figure 1A).

Principal component analysis (PCA) was performed on the SNP and Indel sets of the 258 rice samples based on all obtained SNPs, and the first 10 principal components (PCs) were calculated (Figure 1B). The first two principal components accounted for over 80% of the variation, with PC1 (74.27%) and PC2 (6.32%). According to the distribution pattern of genetic background, there was a clear population differentiation, and all samples could essentially be divided into two subgroups, *Osi* and *Osj*. Among them, 28 Egyptian rice samples were assigned to *Osi*, and another 28 to *Osj*, consistent with the results of the phylogenetic tree. Additionally, we used the admixture software to assess the ancestral components of the 258 accessions (Figure 1C). The analysis indicated that the optimal classification was at K = 7, and the visualization of the population structure clearly displayed the ancestral components of each sample, showing distinct population affiliations: 24 newly sequenced materials were distributed in the ind-1 group, and 26 newly sequenced materials were distributed in the jap1 group.

### 2.2. Genomic Variation

**Figure 2:**
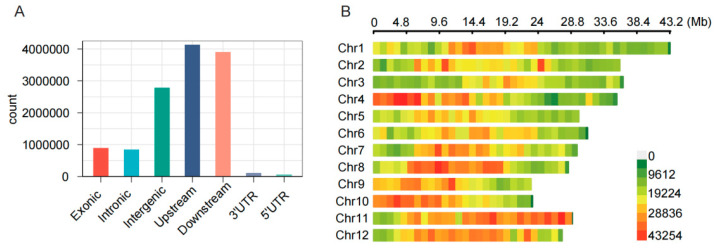
Genome-wide SNP variation statistics. (**A**) Distribution of SNPs across different functional genomic regions; (**B**) SNP density distribution across different chromosomes.

Whole-genome resequencing of the 56 Egyptian rice accessions yielded 614.22 million clean reads, totaling 89.5 Gb of data. An average of 95.02% of the reads were successfully aligned to the reference genome (MSUv7). Our analysis identified a total of 4,410,690 high-quality SNPs and 689,718 indels (Figure 2). Chromosome 1 harbored the highest density of variants (396,001 sites). Functional annotation revealed that 21.71% of variants resided in intergenic regions, 62.9% were located within 2 kb of gene promoters/terminators, and 15.39% were within gene bodies (Figure 2A). Within coding sequences, we observed 487,283 nonsynonymous SNPs and 34,715 frameshift indels. Notably, these variants introduced 21,352 premature stop codons (SNPs) and 1030 (indels), and disrupted the native stop codon in 2010 and 408 cases, respectively (Figure 2B). A comparative analysis with a mini-core collection of 202 Asian cultivated rice accessions identified 18,204 novel SNPs and 2405 novel indels, underscoring the value of this Egyptian germplasm as a unique resource for rice genetics and breeding.

### 2.3. Salinity Tolerance Evaluation

**Figure 3:**
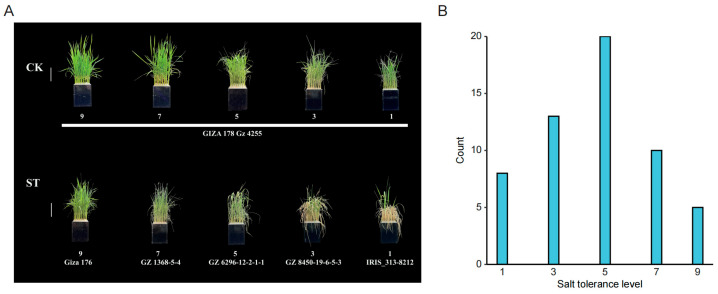
Salt tolerance scores of 56 Egyptian rice accessions. (**A**) Frequency distribution of salt tolerance scores; (**B**) phenotypic characteristics of plants with different salt tolerance scores.

The newly sequenced 56 Egyptian rice varieties provide a valuable resource for discovering novel candidate genes and key genetic variants associated with salt tolerance. In a previous study [24], we performed the GWAS analysis on 202 Asian cultivated rice accessions under salt stress, identifying several candidate genes for this trait. Here, under consistent experimental conditions, we evaluated the salt tolerance of the 56 Egyptian germplasms using the same standardized scoring system (Figure 3A, Table S1). Forty-one of these accessions displayed salt tolerance scores of 5 or higher (Figure 3B), demonstrating a notable level of salt tolerance and underscoring their potential for subsequent genetic analysis.

### 2.4. RNA-Seq and DEGs Analysis

**Figure 4:**
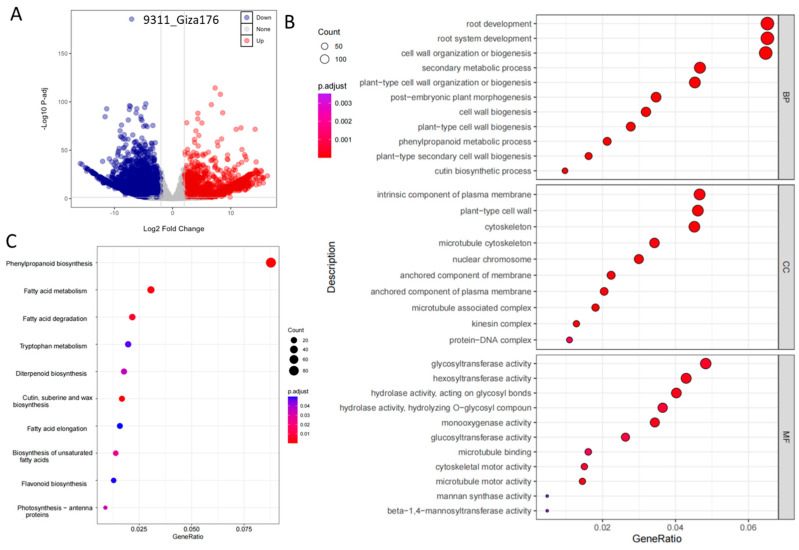
Transcriptome analysis of salt tolerance in Giza 176 and 9311 cultivars. (**A**) DEG analysis of Giza 176 and 9311; (**B**) GO classification and (**C**) KEGG classification of Giza 176 and 9311.

To investigate the transcriptional response mechanism of Egyptian rice to salt stress, we performed RNA-Seq analysis on the salt-tolerant Egyptian landrace Giza 176 and the salt-sensitive model indica rice variety 9311. Giza 176 represents a local salt-tolerant germplasm resource, while 9311 was selected as an ideal control due to its well-defined salt-sensitive phenotype and comprehensive genome annotation. At the seedling stage, plants were treated with the NaCl solution. Samples from both treated and control groups were collected for RNA extraction, library construction, and sequencing. Based on gene expression (FPKM) analysis under salt stress, comparing 9311 with Giza 176 (9311 vs. Giza1), we identified 6504 significantly differentially expressed genes (DEGs), of which 2149 were up-regulated and 4355 were down-regulated (Figure 4A). Compared to normal growth conditions, salt stress induced a more extensive and pronounced transcriptional reprogramming, indicating a significant activation of gene expression reconfiguration.

To further decipher the biological functions of the DEGs under salt stress, we conducted Gene Ontology (GO) enrichment analysis and KEGG pathway annotation on the set of genes specifically expressed in Giza 176. GO analysis revealed that the significantly up-regulated genes in this cultivar were primarily enriched in biological processes closely associated with salt tolerance, including phenylpropanoid biosynthesis, and cutin, suberin, and wax biosynthesis, as well as fatty acid metabolism (Figure 4B). KEGG pathway analysis further indicated significant enrichment of these genes in phenylpropanoid biosynthesis, cutin, suberin and wax biosynthesis, fatty acid metabolism, and fatty acid degradation pathways (Figure 4C). Notably, in Giza 176, multiple pathway genes related to lipid metabolism and secondary metabolite synthesis exhibited more pronounced up-regulation compared to their homologs in 9311. These results demonstrate that Giza 176 establishes its unique salt adaptation mechanism by activating multiple pathways, including phenylpropanoid synthesis, lipid metabolism, and plant hormone signal transduction. The dissection of this regulatory network not only provides new insights into the physiological mechanism of salt tolerance in Egyptian rice landraces but also offers a valuable basis for screening key candidate genes in molecular breeding for salt-tolerant rice.

### 2.5. GWAS and Haplotype Analysis of Candidate Genes

**Figure 5:**
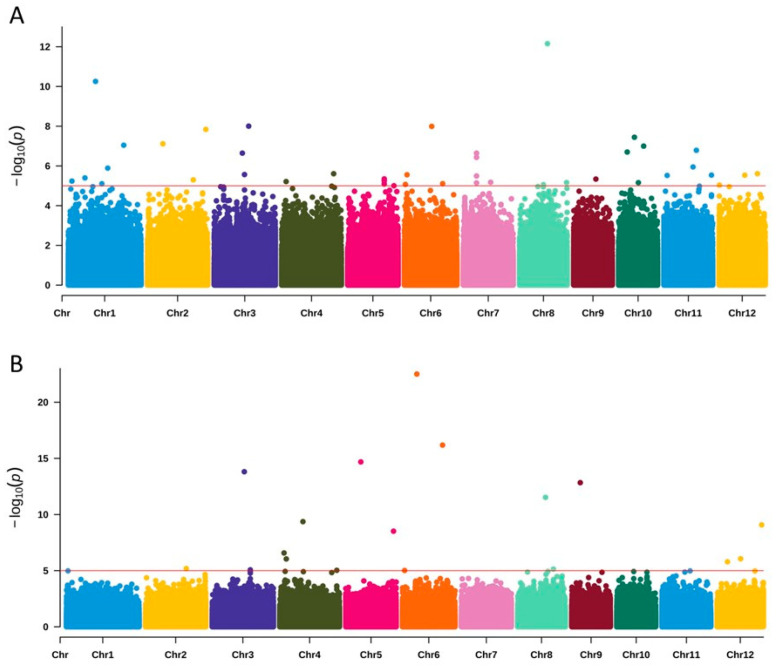
GWAS for salt tolerance identifies novel loci. Manhattan plot of GWAS in (**A**) 202 rice accessions and (**B**) 258 rice accessions. Threshold line is 5.

The GWAS analysis was conducted using the FARMCUP software (v1.02). The GWAS analysis integrated the salt tolerance phenotypes of 202 Asian cultivated rice materials and 56 Egyptian germplasms, enabling us to mine new candidate loci for salt tolerance (Figure 5). The GWAS results showed that the salt tolerance scores of 258 materials identified a total of 19 significant loci, of which 17 were newly identified significant loci (Supplementary Table S2). This suggests that more candidate genes for salt tolerance can be identified.

**Figure 6:**
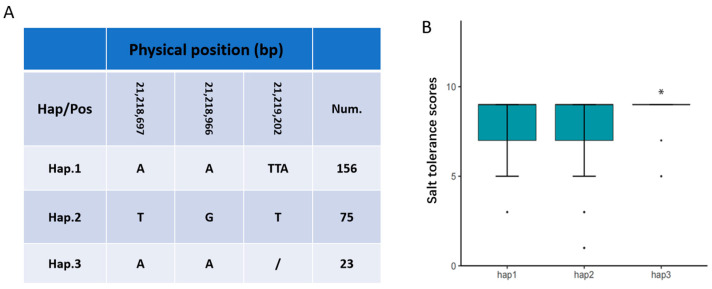
Haplotype analyses of *ONAC063*. Gene structure (**A**) and salt tolerance scores (**B**) of different haplotypes. *t*-test, * *p* < 0.01.

The GWAS analysis provided a novel dataset of candidate genes for salt tolerance, defining 19 candidate QTL intervals. These intervals encompass three previously cloned salt tolerance-related genes: *OsIPK1* (*LOC_Os04g56580*, encoding inositol 1,3,4,5,6-pentakisphosphate 2-kinase) [25], *OsRPH1/STG5* (*LOC_Os05g49700*, encoding an AP2/ERF transcription factor) [23], and *ONAC063* (*LOC_Os08g33910*, encoding a NAC transcription factor) [26]. Furthermore, integrated transcriptomic analysis facilitated additional gene mining, revealing an overlap between the DEGs and candidate genes from GWAS. Specifically, the two datasets co-localized the *ONAC063* gene, which has previously been cloned as a salt-tolerance gene. To comprehensively assess genetic variation within *ONAC063*, we systematically examined all polymorphic sites across 258 rice accessions for potential impacts on the coding sequence. We identified that nucleotide variation within this gene resulted in missense mutations. Therefore, based on salt tolerance phenotypes (Figure 6A), haplotype analysis of *ONAC063*—defined by these missense variant site—across 258 rice accessions revealed multiple haplotypes, with Hap3 identified as a favorable haplotype associated with enhanced salt tolerance (Figure 6B).

### 2.6. Genetic Selection Analysis

**Figure 7:**
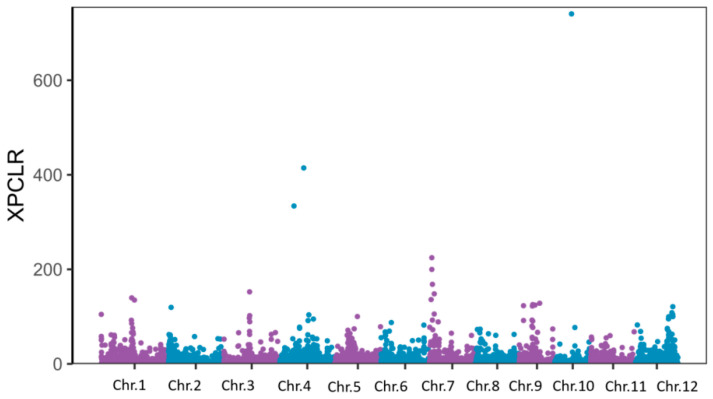
*Fst* analysis highlights genomic differentiation.

To elucidate the genetic basis of salt tolerance variation in Egyptian rice varieties, we performed a population differentiation analysis on 56 Egyptian rice accessions and 202 rice mini-core accessions, applying *Fst* analysis to measure allele frequency divergence between populations and detect genomic regions that may be under selection (Figure 7). Setting the top 5% of *Fst* values as the threshold revealed 17,679 significantly differentiated loci, including 62 annotated salt tolerance genes (Supplementary Table S3). Functional annotation implicated these genes in processes critical to salt tolerance, such as ion transport, osmotic regulation, and antioxidant responses. Several key genes (e.g., *OREB1* [27], *OsHKT1;1* [21], *OsSOS1* [28]) were prioritized. These results indicate that genes within divergent genomic regions have experienced differential selection and likely underpin salt tolerance adaptations, potentially driven by distinct breeding histories or environmental pressures.

## 3. Discussion

Soil salinization poses a major threat to global food security by severely impacting rice, a salt-sensitive crop, thereby making the genetic enhancement of salt tolerance an urgent breeding priority [8,10]. This study provides a comprehensive genomic characterization of a unique panel of 56 Egyptian rice accessions and integrates it with a diverse Asian mini-core collection to elucidate the genetic architecture of salt tolerance. Our findings confirm that the Egyptian germplasm represents a valuable and distinct genetic reservoir, equally divided between the indica and japonica subspecies, and harbors substantial novel genetic variation, including over 20,000 novel SNPs and indels not found in the Asian panel. This underscores the critical importance of exploring locally adapted, non-reference germplasms to fully capture the genetic diversity of cultivated rice.

Salt tolerance in rice is a complex quantitative trait governed by multiple genes, which has been extensively investigated through genetic mapping studies [14]. This study provides a comprehensive genomic characterization of a unique panel of 56 Egyptian rice accessions and integrates it with a diverse Asian mini-core collection to elucidate the genetic architecture of salt tolerance. Our findings confirm that the Egyptian germplasm represents a valuable and distinct genetic reservoir, equally divided between the indica and japonica subspecies, and harbors substantial novel genetic variation, including over 20,000 novel SNPs and indels not found in the Asian panel. This underscores the critical importance of exploring locally adapted, non-reference germplasms to fully capture the genetic diversity of cultivated rice.

The core of our strategy was a multi-faceted genomic analysis. The GWAS conducted on the expanded panel of 258 accessions proved highly effective, identifying 19 significant loci for salt tolerance, 17 of which are novel. This significant increase in discovery power highlights the advantage of incorporating genetically distinct populations like the Egyptian accessions into association studies, which helps to break down linkage disequilibrium and uncover new trait-associated loci. Critically, our integrated transcriptomic analysis provided functional context to these genomic findings. The comparative RNA-Seq of the salt-tolerant cultivar Giza 176 and the sensitive 9311 under stress revealed comprehensive transcriptional reprogramming, with 6504 DEGs identified. The significant enrichment of these DEGs in key pathways such as phenylpropanoid biosynthesis, cutin/suberin/wax deposition, and fatty acid metabolism in the tolerant genotype delineates a robust, multi-faceted defense strategy at the transcriptional level. The validation of our approach is reinforced by the fact that our candidate intervals encompassed known salt-tolerance genes such as *OsIPK1* [25], *STG5* [23], and *ONAC063* [26]. The convergence of GWAS and transcriptomic data was particularly powerful for *ONAC063*, which was not only located within a significant GWAS locus but also emerged as a salt-responsive DEG, providing strong multi-omics support for its central role. The haplotype analysis of *ONAC063* further confirmed its role, identifying a specific haplotype (Hap3) associated with enhanced tolerance, suggesting potential for marker-assisted selection.

Complementary to the GWAS, the population differentiation (*Fst*) and selection sweep (XP-CLR) analyses offered an evolutionary perspective on salt tolerance. The identification of 62 annotated salt-tolerance genes within the highly differentiated genomic regions suggests that the Egyptian germplasm has been subjected to selective pressures, potentially related to its adaptation to local environments, that have shaped its unique genetic makeup. Genes involved in ion homeostasis (e.g., *OsHKT1;1* [21], *OsSOS1* [28]) and osmotic stress response were prominently represented, aligning with the fundamental physiological mechanisms of salt tolerance in plants. Notably, several genes within these selected regions are implicated in similar biological processes (e.g., lipid metabolism and secondary cell wall formation) that were highlighted by our transcriptomic data, suggesting a potential concordance between evolutionary selection and stress-responsive regulation.

This study also has limitations. The phenotypic evaluation, while standardized, was conducted at the seedling stage in a controlled hydroponic system. The genetic mechanisms of tolerance at reproductive stages, which are equally critical for yield, may differ and require further investigation. Furthermore, the transcriptomic analysis, while revealing the defense network in Giza 176, was based on a single tolerant-susceptible pair; expanding this to a diverse set of accessions would capture a broader spectrum of transcriptional strategies. The candidate genes and loci identified here are predominantly based on statistical genetic evidence. Functional validation through mutagenesis or transgenic approaches is an essential next step to confirm the biological role of these genes, particularly the novel loci.

In conclusion, our work demonstrates that Egyptian rice germplasm is a rich and underexplored source of allelic diversity for salt tolerance. By employing an integrated genomic approach, we have successfully delineated a set of high-confidence candidate genes and loci, bridging genomic polymorphisms with stress-responsive transcriptional networks. These findings not only advance our understanding of the polygenic nature of salt tolerance in rice but also provide a practical foundation for molecular breeding programs. The molecular markers and candidate genes identified here can be directly utilized to develop elite cultivars with improved resilience to salinity, contributing to the goal of securing rice production in saline-affected regions worldwide.

## 4. Materials and Methods

### 4.1. Materials and Phenotypic Variation

A total of 56 accessions of Egyptian rice germplasm, conserved at the germplasm bank of the China National Rice Research Institute (CNRRI), were evaluated for salt tolerance using a hydroponic system. The experiments were conducted in the greenhouses at CNRRI, following the previously described methodology. Strict controls were employed to ensure that all seeds were from a single harvest batch, treatment applications were synchronized, and all environmental and experimental conditions were kept uniform [24]. The grains were germinated in water at 37 °C in the dark for 3 days. Seeds were germinated for three days, and the resulting seedlings were grown in a nutrient solution for two weeks. We conducted pre-experiments to determine the appropriate salt stress level and observed that a 100 mM NaCl treatment resulted in the most diverse phenotypic distribution, effectively discriminating accessions with different tolerance levels. The seedlings were cultivated in Yoshida’s culture solution, the composition of which was prepared as described by Jahan et al. [29]. The nutrient solution was renewed every two days. After a 14-day pre-culture period, uniformly growing seedlings from each accession were subjected to salt stress by introducing 100 mM NaCl into the nutrient solution for 14 days. A control group was maintained in the standard nutrient solution without NaCl. The whole experiment was conducted in a randomized complete block design with two independent biological replications for each accession. In each replicate, ten uniformly grown seedlings per accession were evaluated. The experiment was conducted twice for each accession, with ten seedlings evaluated per replicate. Following the salt treatment, the plants were assigned a salt tolerance score based on growth and development phenotypes, according to the Standard Evaluation System (SES) scale of 1, 3, 5, 7, and 9 established by Gregorio et al. [30]. The final result for each accession was calculated as the average across the three replicates. This scoring system effectively discriminated susceptible from tolerant and moderately tolerant genotypes, with lower scores correlating with more severe symptoms such as brown leaf tips, leaf yellowing, drying, and stunted growth.

### 4.2. Variant Calling

DNA from a single plant per accession was sequenced on the DNBSEQ-T7 platform (v0.23.2) (15×coverage) (Mgi Tech Co., Ltd., Shenzhen, China). The bioinformatic processing pipeline included: quality control with Fastp (v0.23.2) [31], alignment to the Nipponbare genome (MSU v7.0) using BWA (v0.7.17-r1188) (mem-M-R-K 10000000), and file handling with Samtools (v1.9) [32]. SNPs were called using the GATK4 HaplotypeCaller pipeline (GVCF mode), followed by joint genotyping [33]. The initial SNP set was filtered with GATK’s VariantFiltration tool using standard quality metrics, and subsequently with VCFtools (maf 0.05 [34], max missing 0.8 to apply a minor allele frequency (MAF) cutoff of 0.05 and remove sites with more than 20% missing data.

### 4.3. Genotype and Population Structure Analysis

The genomic data for this study were sourced from two sets of materials. One comprised 202 Asian cultivated rice accessions with publicly available genotypes [35], and the other consisted of 58 Egyptian accessions sequenced specifically for this project. The resequencing data for the Egyptian rice were processed through the SENTIEON DNAseq pipeline. Following functional annotation of all SNPs using SnpEff, we performed a suite of population genetic analyses [36]. This included PCA implemented in PLINK (v1.90b6.21), population structure assessment with ADMIXTURE [37] and phylogeny reconstruction using IQ-TREE [38]. The final tree was rendered for visualization in iTol (http://itol.embl.de/) (accessed on 2 November 2025).

### 4.4. Fst Analysis and Selected Signal Calculation Method

To identify genetic signatures of selection for salt tolerance in Egyptian rice, we conducted a genome-wide analysis using the cross-population composite likelihood ratio (XP-CLR) test [39]. The analysis was performed with a non-overlapping 10 kb sliding window across the genome. Key parameters included a step size of 100 kb, and a maximum of 500 SNPs per window. Genomic regions with elevated XP-CLR scores are indicative of potential selective sweeps. Consequently, we defined candidate selected regions based on the top 5% of the XP-CLR scores, and all genes located within these regions were considered candidate genes associated with salt tolerance.

### 4.5. Transcriptome Analysis

Transcriptome analysis was conducted on the salt-tolerant cultivar Giza 176 and salt-sensitive cultivar 9311. Seedlings were grown hydroponically and treated with 100 mM NaCl for 14 days. The samples from three experimental replicates were pooled to form three biological replicates per cultivar. Total RNA was extracted from shoot tissues using TRIzol reagent (Invitrogen). Raw reads were processed using fastp (v0.23.2) [23,24].

Clean reads were aligned to the Nipponbare reference genome (MSUv7.0) using HISAT2 (v2.2.1) [40]. Gene expression was quantified using featureCounts (v2.0.1) with parameters: -T 2 -p -t exon -g gene_id [41]. Differentially expression genes (DEGs) analysis was performed using edgeR (v3.40.2) with thresholds: CPM > 1, bcv = 0.2, *p*-value < 0.01, |log_2_FC| ≥ 2 [42]. Genes that were differentially expressed in both biological replicates were defined as core DEGs and subjected to functional annotation via GO and KEGG pathway analyses using TBtools (v2.370) [43].

### 4.6. Whole Genome Association Analysis

Using the FarmCPU model from the R package rMVP (v1.02) for whole genome association analysis. Filter out the high-quality SNPs (missing rate ≤ 0.2; minimum allele frequency MAF ≥ 0.05) [44]. The significance threshold for the association analysis is −lg*P* = 5 [45,46], and genes within ± 200 kb upstream and downstream of the significant SNPs are selected as candidate genes [47].

### 4.7. Haplotype Analysis

When conducting haplotype analysis on target genes, variance analysis in the study employs *t*-tests and Wilcoxon tests using R version 4.3.0, and multiple comparisons are performed using the R package multcomp (version 1.4-25).

### 4.8. Statistical and Visualization

Data were subjected to one-way analysis of variance (ANOVA) to determine significant differences between treatments. Subsequent post hoc analysis was performed using Duncan’s multiple range test. For data visualization, GraphPad Prism 10 was utilized to create bar graphs, while Cloud tools (www.igenebook.com) (accessed on 2 November 2025) were employed to generate Venn diagrams, scatter plots, and heat maps.

## 5. Conclusions

This study establishes Egyptian rice germplasm as a valuable genetic reservoir for salt tolerance breeding. Through an integrated genomic and transcriptomic approach, we identified 17 novel loci and high-confidence candidate genes. Comparative transcriptomics further revealed activated defense pathways in the tolerant genotype, and the convergence of GWAS with transcriptomic data strengthened evidence for key genes like *ONAC063*. These findings expand the genetic landscape of salt tolerance and provide a robust foundation for molecular breeding. Future work will focus on functional validation to develop resilient cultivars for saline-affected regions.

## Data Availability

The original contributions presented in the study are included in the article/Supplementary Materials, further inquiries can be directed to the corresponding author.

## Supplementary Materials

The following supporting information can be downloaded at: https://www.mdpi.com/article/10.3390/plants15010128/s1, Table S1: List of evaluated salt tolerance scores for the 56 rice accessions. Table S2: Summary of significant loci associated with salt tolerance identified in the GWAS; Table S3: Identification of the salt tolerance genes among the Top 5% differentiated Loci.

## References

[1] Chen R., Deng Y., Ding Y., Guo J., Qiu J., Wang B., Wang C., Xie Y., Zhang Z., Chen J. (2022). Rice functional genomics: Decades’ efforts and roads ahead. Sci. China Life Sci..

[2] Jahan N., Zhang Y., Lv Y., Song M., Zhao C., Hu H., Cui Y., Wang Z., Yang S., Zhang A. (2019). QTL analysis for rice salinity tolerance and fine mapping of a candidate locus *qSL7* for shoot length under salt stress. Plant Growth Regul..

[3] Liu M., Pan T., Allakhverdiev S.I., Yu M., Shabala S. (2020). Crop halophytism: An environmentally sustainable solution for global food security. Trends Plant Sci..

[4] Zhao C., Zhang H., Song C., Zhu J.K., Shabala S. (2020). Mechanisms of plant responses and adaptation to soil salinity. Innovation.

[5] Bhatt T., Sharma A., Puri S., Minhas A.P. (2020). Salt tolerance mechanisms and approaches: Future scope of halotolerant genes and rice landraces. Rice Sci..

[6] Ponce K.S., Meng L., Guo L., Leng Y., Ye G. (2021). Advances in sensing, response and regulation mechanism of salt tolerance in rice. Int. J. Mol. Sci..

[7] Morton M.J.L., Awlia M., Al-Tamimi N., Saade S., Pailles Y., Negrao S., Tester M. (2019). Salt stress under the scalpel—Dissecting the genetics of salt tolerance. Plant J..

[8] Amanat M.A., Naeem M.K., Algwaiz H.I.M., Uzair M., Attia K.A., AlKathani M.D.F., Zaid I.U., Zafar S.A., Inam S., Fiaz S. (2022). Evaluation of green super rice lines for agronomic and physiological traits under salinity stress. Plants.

[9] Miller G., Suzuki N., Ciftci-Yilmaz S., Mittler R. (2010). Reactive oxygen species homeostasis and signalling during drought and salinity stresses. Plant Cell Environ..

[10] Mittler R., Vanderauwera S., Suzuki N., Miller G., Tognetti V.B., Vandepoele K., Gollery M., Shulaev V., Van Breusegem F. (2011). ROS signaling: The new wave?. Trends Plant Sci..

[11] Roy S.J., Negrao S., Tester M. (2014). Salt resistant crop plants. Curr. Opin. Biotechnol..

[12] Fukuda A., Nakamura A., Tagiri A., Tanaka H., Miyao A., Hirochika H., Tanaka Y. (2004). Function, intracellular localization and the importance in salt tolerance of a vacuolar Na(^+^)/H(^+^) antiporter from rice. Plant Cell Physiol..

[13] El Mahi H., Perez-Hormaeche J., De Luca A., Villalta I., Espartero J., Gamez-Arjona F., Fernandez J.L., Bundo M., Mendoza I., Mieulet D. (2019). A critical role of sodium flux via the plasma membrane Na(^+^)/H(^+^) exchanger SOS1 in the salt tolerance of rice. Plant Physiol..

[14] Ganie S.A., Wani S.H., Henry R., Hensel G. (2021). Improving rice salt tolerance by precision breeding in a new era. Curr. Opin. Plant Biol..

[15] Fan X., Jiang H., Meng L., Chen J. (2021). Gene mapping, cloning and association analysis for salt tolerance in rice. Int. J. Mol. Sci..

[16] Huang X., Feng Q., Qian Q., Zhao Q., Wang L., Wang A., Guan J., Fan D., Weng Q., Huang T. (2009). High-throughput genotyping by whole-genome resequencing. Genome Res..

[17] Huang X., Kurata N., Wei X., Wang Z.X., Wang A., Zhao Q., Zhao Y., Liu K., Lu H., Li W. (2012). A map of rice genome variation reveals the origin of cultivated rice. Nature.

[18] Huang X., Wei X., Sang T., Zhao Q., Feng Q., Zhao Y., Li C., Zhu C., Lu T., Zhang Z. (2010). Genome-wide association studies of 14 agronomic traits in rice landraces. Nat. Genet..

[19] Yano K., Yamamoto E., Aya K., Takeuchi H., Lo P.C., Hu L., Yamasaki M., Yoshida S., Kitano H., Hirano K. (2016). Genome-wide association study using whole-genome sequencing rapidly identifies new genes influencing agronomic traits in rice. Nat. Genet..

[20] Huang X.Y., Chao D.Y., Gao J.P., Zhu M.Z., Shi M., Lin H.X. (2009). A previously unknown zinc finger protein, DST, regulates drought and salt tolerance in rice via stomatal aperture control. Genes Dev..

[21] Ren Z.H., Gao J.P., Li L.G., Cai X.L., Huang W., Chao D.Y., Zhu M.Z., Wang Z.Y., Luan S., Lin H.X. (2005). A rice quantitative trait locus for salt tolerance encodes a sodium transporter. Nat. Genet..

[22] Deng P., Jing W., Cao C., Sun M., Chi W., Zhao S., Dai J., Shi X., Wu Q., Zhang B. (2022). Transcriptional repressor RST1 controls salt tolerance and grain yield in rice by regulating gene expression of asparagine synthetase. Proc. Natl. Acad. Sci. USA.

[23] Wei H., Wang X., Zhang Z., Yang L., Zhang Q., Li Y., He H., Chen D., Zhang B., Zheng C. (2024). Uncovering key salt-tolerant regulators through a combined eQTL and GWAS analysis using the super pan-genome in rice. Natl. Sci. Rev..

[24] Lv Y., Ma J., Wei H., Xiao F., Wang Y., Jahan N., Hazman M., Qian Q., Shang L., Guo L. (2022). Combining GWAS, genome-wide domestication and a transcriptomic analysis reveals the loci and natural alleles of salt tolerance in rice (*Oryza sativa* L.). Front. Plant Sci..

[25] Jiang M., Liu Y., Li R., Li S., Tan Y., Huang J., Shu Q. (2020). An inositol 1,3,4,5,6-Pentakisphosphate 2-Kinase 1 mutant with a 33-nt deletion showed enhanced tolerance to salt and drought stress in rice. Plants.

[26] Yokotani N., Ichikawa T., Kondou Y., Matsui M., Hirochika H., Iwabuchi M., Oda K. (2009). Tolerance to various environmental stresses conferred by the salt-responsive rice gene *ONAC063* in transgenic Arabidopsis. Planta.

[27] Li Y., Zhou J., Li Z., Qiao J., Quan R., Wang J., Huang R., Qin H. (2022). SALT AND ABA RESPONSE ERF1 improves seed germination and salt tolerance by repressing ABA signaling in rice. Plant Physiol..

[28] Martinez-Atienza J., Jiang X., Garciadeblas B., Mendoza I., Zhu J.K., Pardo J.M., Quintero F.J. (2007). Conservation of the salt overly sensitive pathway in rice. Plant Physiol..

[29] Jahan N., Lv Y., Song M., Zhang Y., Shang L., Lu Y., Ye G., Qian Q., Gao Z., Guo L. (2021). Transcriptomic analysis of short-term salt-stress response in mega hybrid rice seedlings. Agronomy.

[30] Gregorio G.B., Senadhira D., Mendoza R.D. (1997). Screening Rice for Salinity Tolerance.

[31] Chen S. (2023). Ultrafast one-pass FASTQ data preprocessing, quality control, and deduplication using fastp. Imeta.

[32] Li H., Handsaker B., Wysoker A., Fennell T., Ruan J., Homer N., Marth G., Abecasis G., Durbin R., Genome Project Data Processing S. (2009). The sequence alignment/map format and SAMtools. Bioinformatics.

[33] Heldenbrand J.R., Baheti S., Bockol M.A., Drucker T.M., Hart S.N., Hudson M.E., Iyer R.K., Kalmbach M.T., Kendig K.I., Klee E.W. (2019). Recommendations for performance optimizations when using GATK3.8 and GATK4. BMC Bioinform..

[34] Danecek P., Auton A., Abecasis G., Albers C.A., Banks E., DePristo M.A., Handsaker R.E., Lunter G., Marth G.T., Sherry S.T. (2011). The variant call format and VCFtools. Bioinformatics.

[35] Shang L., Li X., He H., Yuan Q., Song Y., Wei Z., Lin H., Hu M., Zhao F., Zhang C. (2022). A super pan-genomic landscape of rice. Cell Res..

[36] Kawahara Y., de la Bastide M., Hamilton J.P., Kanamori H., McCombie W.R., Ouyang S., Schwartz D.C., Tanaka T., Wu J., Zhou S. (2013). Improvement of the Oryza sativa Nipponbare reference genome using next generation sequence and optical map data. Rice.

[37] Alexander D.H., Lange K. (2011). Enhancements to the ADMIXTURE algorithm for individual ancestry estimation. BMC Bioinform..

[38] Minh B.Q., Schmidt H.A., Chernomor O., Schrempf D., Woodhams M.D., von Haeseler A., Lanfear R. (2020). IQ-TREE 2: New models and efficient methods for phylogenetic inference in the genomic era. Mol. Biol. Evol..

[39] Chen H., Patterson N., Reich D. (2010). Population differentiation as a test for selective sweeps. Genome Res..

[40] Kim D., Langmead B., Salzberg S.L. (2015). HISAT: A fast spliced aligner with low memory requirements. Nat. Methods.

[41] Trapnell C., Roberts A., Goff L., Pertea G., Kim D., Kelley D.R., Pimentel H., Salzberg S.L., Rinn J.L., Pachter L. (2012). Differential gene and transcript expression analysis of RNA-seq experiments with TopHat and Cufflinks. Nat. Protoc..

[42] Robinson M.D., McCarthy D.J., Smyth G.K. (2010). edgeR: A Bioconductor package for differential expression analysis of digital gene expression data. Bioinformatics.

[43] Chen C., Chen H., Zhang Y., Thomas H.R., Frank M.H., He Y., Xia R. (2020). TBtools: An Integrative Toolkit Developed for Interactive Analyses of Big Biological Data. Mol. Plant.

[44] Visscher P.M., Brown M.A., McCarthy M.I., Yang J. (2012). Five years of GWAS discovery. Am. J. Hum. Genet..

[45] Gao H., Zhang C., He H., Liu T., Zhang B., Lin H., Li X., Wei Z., Yuan Q., Wang Q. (2020). Loci and alleles for submergence responses revealed by GWAS and transcriptional analysis in rice. Mol. Breed..

[46] Lv Y., Ma J., Wang Y., Wang Q., Lu X., Hu H., Qian Q., Guo L., Shang L. (2021). Loci and natural alleles for low-nitrogen-induced growth response revealed by the genome-wide association study analysis in rice (*Oryza sativa* L.). Front. Plant Sci..

[47] Kang H.M., Sul J.H., Service S.K., Zaitlen N.A., Kong S.Y., Freimer N.B., Sabatti C., Eskin E. (2010). Variance component model to account for sample structure in genome-wide association studies. Nat. Genet..

